# Evaluation of Mental Health Emergency Centre Admissions at Northwick Park Hospital

**DOI:** 10.1192/bjo.2026.11646

**Published:** 2026-06-30

**Authors:** Sirous Golchinheydari, Ruben Perumal, Alex Thomson, Khalil Hassanally

**Affiliations:** 1 South London and Maudsley NHS Foundation Trust, London, United Kingdom; 2 East London NHS Foundation Trust, London, United Kingdom; 3 Central and North West London NHS Foundation Trust, London, United Kingdom

## Abstract

**Aims:**

The Mental Health Emergency Centre (MHEC) at Northwick Park Mental Health Unit serves people who present in an acute mental health crisis, most commonly via the emergency department (ED). The MHEC receives several patients a day. To inform service planning, we aimed to describe patient characteristics and clinical interventions delivered.

**Methods:**

The study was approved as a service evaluation by the clinical governance department. Records of patients admitted to the MHEC from February 15, 2023, to October 26, 2023, were reviewed. Using a predefined template, two psychiatry residents extracted information from the records of 50 patients each, including patient demographics, attendance, diagnoses, comorbidities, and reattendance to the ED within 28 days. Incomplete records were excluded.

**Results:**

Ninety-six records were included; 94% came via the ED pathway and 6% admitted directly by the Home Treatment Team (HTT). The mean age was 38 years and 58% of patients were men. The most common ethnicity was white (42%) followed by Asian (18%). The most common primary diagnoses were psychotic disorders (39%). The median time from ED arrival to a decision to admit to the MHEC was 11.8 hours, and the median time from ED arrival to discharge from the MHEC was 62.4 hours. The median length of stay (LOS) from the decision to admit until discharge from the MHEC was 2 days. Reattendance to the ED within 28 days was seen in 28% of patients. Medication management was the main intervention offered (62.5%), followed by brief psychological intervention by a trained psychologist (23%). Only four patients did not have a follow-up.

**Conclusion:**

The MHEC is a valuable addition to the urgent and emergency mental health care pathway. Although the patients were not rapidly transferred from ED, the multidisciplinary service allowed brief and intense interventions to be delivered, including psychological therapies, diagnostic review, and medication management.

